# Stingless Bee Propolis: New Insights for Anticancer Drugs

**DOI:** 10.1155/2021/2169017

**Published:** 2021-09-23

**Authors:** Jaqueline Ferreira Campos, Helder Freitas dos Santos, Thaliny Bonamigo, Nelson Luís de Campos Domingues, Kely de Picoli Souza, Edson Lucas dos Santos

**Affiliations:** ^1^Research Group on Biotechnology and Bioprospecting Applied to Metabolism (GEBBAM), Federal University of Grande Dourados, Dourados, MS, Brazil; ^2^Organic Catalysis and Biocatalysis Laboratory (LACOB), Federal University of Grande Dourados, Dourados, MS, Brazil

## Abstract

Natural products are important sources of biomolecules possessing antitumor activity and can be used as anticancer drug prototypes. The rich biodiversity of tropical and subtropical regions of the world provides considerable bioprospecting potential, including the potential of propolis produced by stingless bee species. Investigations of the potential of these products are extremely important, not only for providing a scientific basis for their use as adjuvants for existing drug therapies but also as a source of new and potent anticancer drugs. In this context, this article organizes the main studies describing the anticancer potential of propolis from different species of stingless bees with an emphasis on the chemical compounds, mechanisms of action, and cell death profiles. These mechanisms include apoptotic events; modulation of BAX, BAD, BCL2-L1 (BCL-2 like 1), and BCL-2; depolarization of the mitochondrial membrane; increased caspase-3 activity; poly (ADP-ribose) polymerase (PARP) cleavage; and cell death induction by necroptosis via receptor interacting protein kinase 1 (RIPK1) activation. Additionally, the correlation between compounds with antioxidant and anti-inflammatory potential is demonstrated that help in the prevention of cancer development. In summary, we highlight the important antitumor potential of propolis from stingless bees, but further preclinical and clinical trials are needed to explore the selectivity, efficacy, and safety of propolis.

## 1. Introduction

Cancer is among the main diseases affecting humanity, accounting for one in six deaths worldwide [1]. Many studies have been conducted to search for anticancer drugs that control and/or inhibit the proliferation of cancer cells. From 1946 to 2019, 321 anticancer drugs were approved by the Food and Drug Administration (FDA), 168 (52.3%) of which were related to natural products: 35 were of natural origin, 65 were derived from natural products, 45 were mimics of natural products, 22 had pharmacophores obtained from natural products, and one contained plant matter as an ingredient [2].

Some of the first anticancer drugs derived from natural products approved for clinical use include vincristine and vinblastine, which are both alkaloids obtained from *Catharanthus roseus* (L.) G. Don; camptothecin, an alkaloid obtained from *Camptotheca acuminata Decne*; and paclitaxel, a diterpene originally isolated from the bark of *Taxus brevifolia* Nutt., all of which are of plant origin [3, 4]. These drugs are still used today, and the scientific literature details both the clinical importance of their uses [5–7] and their mechanisms of action [8, 9].

Additionally, other preclinical and clinical studies are being conducted with different drugs derived from phenolic compounds with anticancer potential, such as alvocidib, a synthetic flavonoid approved in phase 2 clinical studies for the treatment of different types of cancer, such as prostate cancer, pancreatic cancer, and leukemias; genistein, an isoflavonoid derived from soybean approved in phase 2 studies for the prevention and/or treatment of breast cancer and adenocarcinoma; and its synthetic derivative, idronoxil, which has been approved in phases 2 and 3 of clinical trials for the treatment of prostate and ovarian cancers, respectively [10].

Alkaloids, terpenes, and phenolic compounds are bioactive compounds with anticancer potential, are found mainly in plants, and are concentrated in microorganisms and metabolites of animal origin. In this context, research on the development of new anticancer drugs has not been limited only to plant substances or molecules. Researchers have investigated the anticancer potential of compounds produced by endophytic fungi [11], wasp venom peptides [12], and ant venom [13], as well as natural products from different bee species, such as honey [14, 15] and propolis [16, 17].

Among bee products, the propolis produced by the species *Apis mellifera* (Apidae, Apinae) has been extensively studied [18–20] as an antioxidant [21, 22], immunomodulatory agent [23, 24], and antimicrobial agent [25, 26] and mainly used in the prevention and treatment of cancer [17, 27–30]. In a search conducted on August 15, 2021, in the PubMed database of the NCBI (National Center for Biotechnology Information) using the terms “Apis propolis” and “Stingless Bee propolis,” 719 studies were found on the bee propolis of *A. mellifera*, while only 86 studies involved propolis from stingless bees [31], also known as meliponines since they belong to the subfamily Meliponini of the family Apidae [32].

In the tropical and subtropical regions of the world, approximately 600 species of meliponines have been identified [33], most of which produce propolis in addition to other natural products with considerable bioprospecting potential. The anticancer potential of honey [15], cerumen [34], geopropolis [35], and propolis [16, 36, 37] produced by stingless bees has been described in the literature.

The first studies of the chemical composition and biological potential of propolis from meliponines emerged in the 1990s [38, 39]. A few years later, Bankova *et al.* [40] and Bankova and Popova [41] published two reviews on promising natural product. Recently, Lavinas *et al*. [42] published a review on the antioxidant, antimicrobial, and toxic activities of propolis from several species of stingless bees found in Brazil, and Popova *et al*. [43] highlighted the advances related to the chemical composition and biological activities of propolis of species found in the Americas, Asia, and Australia.

Studies developed in the last 30 years describe different mechanisms of action for this natural product and provide data on its safety, which may guide future studies and perspectives. This review highlights the importance of propolis from stingless bees as a promising natural resource for the development of new antitumor drugs and introduces new possibilities from a comparative perspective regarding the chemical substances described in propolis extracts, cell death profiles, and mechanisms of action.

## 2. Propolis: Therapeutic Potential

Propolis is a resinous material produced by bees using plant sources, such as exudates from leaves, stems, and flowers, mixed with wax and mandibular secretions [44, 45]. This material is used by bees to protect the hive from physical and biological factors. Propolis is deposited at the entrance of the nest and in external cracks to prevent invasion by other organisms, on the inside walls of the hive to maintain the appropriate internal temperature, and at the egg-laying site to maintain asepsis and is used to embalm dead organisms inside the nest, preventing the proliferation of microorganisms [46].

For centuries, humans have identified the importance of this product for bees and correlated its use with human health. Egyptians used propolis for its antiputrefactive properties to embalm the dead; Greeks and Romans used propolis as an antiseptic and healing agent; Incas used propolis as an antipyretic agent, and in the 17th century, propolis was listed as an official drug in the London Pharmacopoeia [47, 48].

During World War II (1939-1945), propolis was widely used as a healing agent and was prescribed by doctors to wounded soldiers [49]. This natural product gained popularity in Europe between the 17th and 20th centuries and was accepted in human and veterinary medicine in 1969 in the Soviet Union (USSR) for several applications, including the treatment of tuberculosis [45].

In 1985, propolis was recognized as a promising product in pharmacology in Japan [45]. Currently, the Japanese population highly values the benefits of this bee product and is even one of the largest importers of raw material produced in Brazil [48].

In recent decades, scientific publications on the chemical composition and biological properties of propolis from different bee species and different geographic origins have significantly increased. Propolis produced by stingless bees possesses antimicrobial [50, 51], antioxidant [52, 53], anti-inflammatory [54, 55], and antitumor [16, 36, 56, 57] properties, among others.

The medicinal properties of propolis are directly related to its chemical composition. Studies conducted with propolis from stingless bees describe the presence of phenolic acids, aromatic acids, terpenes, carbohydrates [53, 58, 59], and alkaloids [16, 57]. The bee species and the botanical source from which they collect resin are among the factors responsible for the physical, chemical, and biological characteristics of this natural product [52, 60].

## 3. Compounds with Antitumor Potential

The diversity of the types of propolis produced by bees from tropical and subtropical regions results from the mixture of different compounds of plant and bee origin. In general, propolis is composed of approximately 50% resin, 30% wax, 10% essential oils, 5% pollen, and 5% other substances, including phenolic compounds, terpenes, aromatic aldehydes, alcohols, fatty acids, and minerals [61].

The genera of stingless bees most studied to determine the chemical composition and biological activities of propolis include *Melipona*, *Plebeia*, *Trigona*, *Scaptotrigona*, *Trigonisca*, and *Tetragonisca* [41, 42]. Bankova and Popova [41] report that the main chemical constituents present in propolis produced by different species of meliponines are terpenes, especially di- and triterpenes, in addition to phenolic acids and sugars. Sanches *et al*. [60] emphasize that stingless bees are attracted to terpenes and enrich propolis with raw material rich in these compounds.

Terpenes are compounds commonly found in different types of propolis, and several studies have shown their anticancer potential [62, 63]. The mechanisms of action evidenced for these compounds include the modulation of the p53, peroxisome proliferator-activated receptor gamma (PPAR*γ*), mitogen-activated protein kinase (MAPK), nuclear factor-*κ*B (NF-*κ*B), phosphoinositide 3-kinase (PI3K)/protein kinase B (AKT), and signal transducer and activator of transcription 3 (STAT3) signaling pathways and disruption of microtubules in the mitotic spindle, the latter of which is the main mechanism underlying the cytotoxic action of paclitaxel, one of the most successful commercially available terpenes [64].

In India, when investigating the composition of propolis produced by *Trigona* sp., Choudhari *et al*. [51] identified the presence of 24 compounds, including terpenes, alkanes, thiophilic acids, aromatic acids, aliphatic acids, sugars, and esters. Studies conducted with propolis from different bee species found in the Cerrado biome in the Central-West region of Brazil reported the presence of different proportions of terpenes, phenolic compounds, aromatic acids, and sugars in the ethanolic extracts of propolis from *Melipona orbignyi* [58] and *Tetragonisca fiebrigi* [55], which can directly influence the biological activities investigated.

When comparing the propolis of *Melipona quadrifasciata anthidioides* and *Scaptotrigona depilis*, Bonamigo *et al*. [36] observed that both extracts contain terpenes, phytosterols, tocopherol, and phenolic compounds at different concentrations and noted that only the extract of *M. q. anthidioides*, which showed the best cytotoxic action, contains the phenolic compounds vanillic acid, caffeic acid, quercetin, luteolin, and apigenin in its composition.

**Figure 1:**
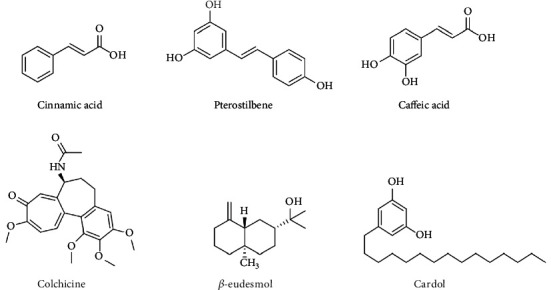
Compounds found in propolis extracts from stingless bees that are promising for the development of antitumor drugs.

Globally, Brazil leads other countries in the number of studies assessing the chemical composition and biological activities of propolis from different species of stingless bees [42]. The country has substantial biodiversity, which contributes to the complexity of the types of this bee product. In southern Brazil, Cisilotto *et al*. [57] showed the unusual presence of piperidine alkaloids in the propolis extract of *Scaptotrigona bipunctata*, and in the Philippines, Desamero *et al*. [16] highlighted that the propolis of the species *Tetragonula biroi* contains more than 500 chemical constituents; approximately 15 of these compounds are promising candidates with anticancer activity according to chemical structure analyses and previous reports in the literature, including phenolic compounds (cinnamic acid and pterostilbene), terpenoids (*β*-eudesmol), and alkaloids (colchicine), represented in Figure 1.

Colchicine has anticancer effects, induces caspase 3-mediated apoptosis via suppressing the PI3K/Akt/mTOR signaling pathway on human gastric cancer cell lines, and suppresses the tumor growth in vivo [65]. Alkaloids are molecules widely known to have antitumor potential, and their main mechanisms of action include inhibition of the polymerization of microtubules that bind to *β*-tubulin subunits, inhibition of protein synthesis, and blockade of cell cycle progression, resulting in cell death by apoptosis [8].

Among the phenolic compounds, cinnamic acid was also found in propolis from *Tetragonisca fiebrigi* [55] and is considered one of the most potent antitumors, besides acting in the biosynthetic route of phenolic acids, such as caffeic acid (Figure 1), that acts on cell cycle modulation, inhibition of colony formation, and proapoptotic properties in cancer cells [66]. Pterostilbene, the 3,5-dimethoxy motif at the A-phenyl ring of resveratrol, has presented chemopreventive and chemotherapeutic properties in breast, prostate, and colon cancer cells, by inducing apoptotic and autophagic pathways, regulating the expression of metastasis-related proteins [67].

## 4. Cytotoxic Potential of Propolis against Cancer Cell Lines

The anticancer activity of an extract, fraction, or compound is evaluated *in vitro* and is initially investigated through the culture of cancer cell lines exposed to the material under study. Propolis from various species of stingless bees from different regions of the world has shown significant anticancer activity in different cell lines, as summarized chronologically in Table 1.

Among the various species, the propolis from the species *M. orbignyi*, *T. fiebrigi*, *S. depilis*, *M. q. anthidioides*, and *Plebeia droryana*, which are found in Brazil, exhibited anticancer potential in human chronic myeloid leukemia K562 cells [36, 55, 58, 77]. Propolis from the species *S. bipunctata* and *M. q. anthidioides* from southern Brazil, state of Santa Catarina, promoted cytotoxicity and inhibited migration and invasion in the human melanoma cell model SK-MEL-28 [57].

In Thailand, Umthong *et al*. [68] reported the antiproliferative activity of *Trigona laeviceps* propolis against five human cancer cell lines, namely, ductal carcinoma (BT474), colon adenocarcinoma (SW620), liver cancer (HepG2), lung cancer (ChaGo), and gastric cancer (KATO-III). Utispan *et al*. [37] also documented the cytotoxic action of propolis from the species *Trigona sirindhornae* in head and neck cancer cells (HN30). Additionally, propolis from the stingless bees *Tetragonula pagdeni*, *Lepidotrigona ventralis*, and *Lepidotrigona terminata* induced death in four human cancer cell lines, including colorectal cancer (Caco-2), liver cancer (Hep-G2), melanoma (SK-MEL-28), and papilloma carcinoma KB cells [78].

In Indonesia, Kustiawan *et al*. [69] found that ductal carcinoma (BT474), colon adenocarcinoma (SW620), liver cancer (HepG2), lung cancer (ChaGo), and gastric cancer (KATO-III) cell lines were susceptible to the cytotoxic action of propolis from the species *Timia apicalis*, *Trigona fuscobalteata*, *Trigona fuscibasis*, and *Trigona incisa*. In another study, Kustiawan *et al*. [71] showed that propolis from the species *T. incisa* also exerts an antiproliferative effect on colon adenocarcinoma cells (SW620). Iqbal *et al*. [74] showed the cytotoxic action of propolis from *Trigona* spp. in a breast cancer cell line (MDA-MB-231) and documented its antiangiogenic action, while Amalia *et al*. [75] reported the antiproliferative activity of *Trigona* spp. in the MCF-7 breast cancer line.

In Vietnam, the cytotoxic potential of *Trigona minor* propolis was confirmed in a pancreatic cancer cell line (PANC-1) [72]. Propolis from species of the genus *Trigona*, which are common in India, was cytotoxic to breast cancer (MCF-7), colon adenocarcinoma (HT-29), colorectal adenocarcinoma (Caco-2), and murine melanoma (B16F1) cells [56]. Kothai and Jayanthi [70] revealed the cytotoxicity of propolis from the species *Tetragonula iridipennis* toward lung cancer cells (A549). In Malaysia, Mohd-Yazid *et al*. [73] reported that the propolis of the species *Heterotrigona itama*, which was collected at different locations, exerted a moderate cytotoxic effect on cervical carcinoma cells (HeLa), and Mohamed *et al*. [76] showed that the propolis extract of *Tetrigona apicalis* was toxic to breast cancer cells (MCF-7). Additionally, in the Philippines, propolis of the species *T. biroi* decreased the proliferation of four human gastric cancer lines (SFA, MKN-45, NUGC-4, and MKN-74) [16].

## 5. Mechanisms of Cell Death Induced by Propolis

Several cell death mechanisms have been identified based on morphological and molecular parameters, including apoptosis and necrosis [79], which are common mechanisms stimulated by propolis extracts from stingless bees. Apoptosis is a regulated mechanism of cell death performed in a safe and controlled manner by cells [17]. In this process, cells undergo rapid structural and biochemical changes, including nuclear chromatin condensation, cytoplasmic shrinkage, nuclear fragmentation, and the formation of plasma membrane blebs [80, 81]. Finally, the cell decomposes into apoptotic bodies, which are recognized and engulfed by phagocytes [82].

In contrast, necrosis is an energy-independent mechanism in which the cell suffers severe damage caused by a sudden shock, such as exposure to high temperatures, radiation, hypoxia, mechanical damage, and chemical substances, leading to the loss of its functions [83, 84]. During this accidental and unregulated process, cell swelling, rupture of the membranes of the cellular organelles, chromatin digestion, DNA hydrolysis, and cell lysis occur [79]. Rupture of the plasma membrane and extravasation of the cell contents trigger inflammatory cascades [84].

In cases of an absence or unavailability of phagocytes during apoptosis, the process of secondary necrosis may be triggered [85, 86], which is also known as late apoptosis [87]. In addition, for two decades, this type of death was considered an accidental and unregulated process triggered by physicochemical injuries [88, 89]. However, the mechanisms of a regulated form of necrosis, known as necroptosis, have already been described in the literature [90].

Necroptosis is a metabolically active form of regulated cell death resulting in a necrotic morphology [91] characterized by organelle swelling, loss of integrity, and rupture of the plasma membrane [92]. Similar to apoptosis, necroptosis can be induced by cell death receptors, such as *tumor necrosis factor receptor* (TNFR), and external and internal viral stimuli recognized by *Toll-like* receptors under conditions of caspase 8 inhibition [93].

Tumor necrosis factor receptor (TNFR) stimulation accompanied by caspase 8 inactivity activates receptor interacting protein kinase 1 (RIPK1) (RIPK1), which phosphorylates RIPK3 and leads to the formation of a signaling complex known as a necrosome [94–96]. Once activated, RIPK3 recruits and phosphorylates mixed lineage kinase domain-like pseudokinase (MLKL), which translocates to the plasma membrane and promotes cell lysis [93]. Studies have shown that MLKL leads to membrane disruption through the influx of calcium ions [97]. Reactive oxygen species (ROS) may also be involved and contribute to the necroptosis process [98].

The discovery of necroptosis as an alternative form of programmed cell death is advantageous in the treatment of cancer because it involves a highly specialized pathway, enabling specific targeting by drugs [99]. In the human body, programmed cell death (apoptosis) has the physiological role of eliminating abnormal or harmful cells and serves as the main mechanism of tumor suppression. In most cancers, dysregulation of different molecules controls this event [17], which hinders cell death. The main objective of an anticancer agent is to reverse this process, thus eliminating tumor cells. Studies with propolis from different species of stingless bees have reported its ability to induce necrosis and apoptosis in different cancer cell lines (Table 2).

In Brazil, propolis from the species *M. orbignyi*, *T. fiebrigi*, *S. depilis*, and *M. q. anthidioides* showed similar cell death profiles and was able to promote cell death by necrosis and late apoptosis in K562 cells (human leukemia) [36, 55, 58]. In addition, Bonamigo *et al*. [77] preincubated K562 cells with the necroptosis inhibitor necrostatin-1 (NEC-1), followed by treatment with *P. droryana* propolis, and observed a reversal of cell death, confirming that the mechanism of death was mediated by necroptosis, a form of regulated necrosis.

In Indonesia, Kustiawan *et al*. [100] observed that the fractionated propolis extract from *T. incisa* promoted cell death by apoptosis of SW620 cells after 2-6 h of treatment, and after 24-72 h, death by inducing the necrosis was observed. When they isolated the compound cardol (5-pentadecylresorcinol) from this extract, Kustiawan *et al*. [71] confirmed the apoptotic death of the same cell line. The chemical structure of the cardol is represented in Figure 1.

**Figure 2:**
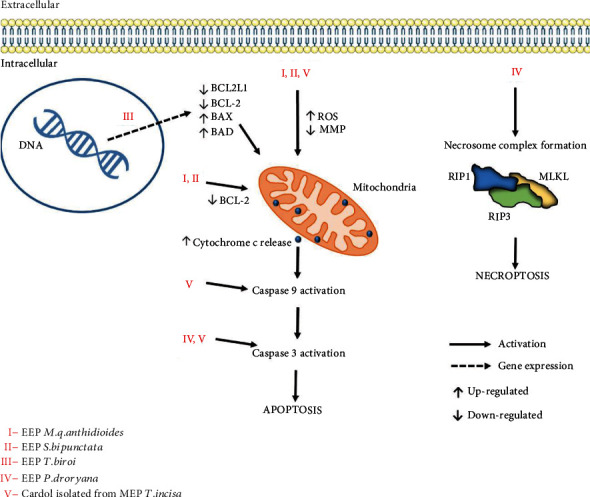
Cell death mechanisms induced by the extracts and/or compounds isolated from propolis from stingless bees.

In comparison, the propolis extract of the species *H. itama* found in Malaysia [73] and the species *S. bipunctata* and *M. q. anthidioides* found in southern Brazil [57] promoted death by apoptosis in HeLa and human melanoma cells (SK-MEL-28), respectively. In a more recent study conducted by Desamero *et al*. [16], apoptotic death was also induced by propolis from the species *T. biroi*, which is found in the Philippines, in the gastric cancer lines (SFA, MKN-45, and NUGC-4). The types of cell death triggered by different propolis extracts from stingless bees are outlined in Figure 2 to better understand and visualize this information.

## 6. Effects of Propolis on the Activation of Apoptotic Pathways

Cell death by apoptosis is activated by the extrinsic pathway through death receptors present on the cell surface or the intrinsic pathway through damage to the mitochondria [101]. The extrinsic pathway is initiated through the interaction of ligands of immune cells with programmed death receptors, such as *TNF receptor 1* (TNFR1), CD95 (also known as Fas and APO-1), *Death receptor 3* (DR3), and *TNF-related apoptosis-inducing ligand receptor 1* (TRAIL-R1), also known as *Death receptor 4* or DR4, while the intrinsic or mitochondrial pathway is triggered by cytotoxic stimuli such as radiation, hypoxia, and DNA damage [84].

Initially, intracellular proteolytic enzymes, known as caspases, which are classified into initiators and effectors, are activated in this cell death pathway [83]. Regarding the extrinsic and intrinsic pathways, the initiator caspases are 8 and 9, respectively [84]. After the activation of caspase 8 or 9, both pathways promote the cleavage and activation of effector caspase 3, resulting in cascades of fragmentation and degradation of DNA and proteins and the formation of apoptotic bodies [102]. Thus, the extrinsic and intrinsic pathways are not completely independent of each other; they converge in the activation of caspases during the signaling cascade, promoting the formation of apoptotic bodies and their engulfment by phagocytosis [103].

Some authors have investigated the role of propolis in inducing apoptotic cell death in more detail (Table 2). In a study conducted by Bonamigo *et al*. [77], in addition to propolis from the species *P. droryana* mainly inducing death by necroptosis, the extract also promoted death by apoptosis, as evidenced by an increase in caspase 3 cleavage and activation in K562 human leukemia cells.

Other molecular targets related to apoptotic pathways can be studied, including initiator caspases. The intrinsic pathway, for example, is controlled by three structurally distinct groups of the BCL protein family: (I) BH3 proteins, which are stimulators; i.e., they transmit signals for the onset of apoptosis; (II) BLC-2 proteins, which promote antiapoptotic action, thus ensuring cell survival; and (III) proapoptotic effector proteins BAX and BAK [82].

The propolis of the bee species found in Brazil, *S. bipunctata* and *M. q. anthidioides*, promoted a decrease in the expression levels of the antiapoptotic protein BCL-2, an increase in ROS production, and depolarization of the mitochondrial membrane, leading to cell death by apoptosis through the modulation of the intrinsic pathway [57]. Similarly, Desamero *et al*. [16] observed that propolis of the species *T. biroi* induces apoptosis by modulating the transcript levels of genes related to the expression of proteins associated with apoptosis. The study revealed a considerable reduction in the expression of the antiapoptotic genes BCL2L1 and BCL-2 and overexpression of the proapoptotic genes BAX and BAD.

The compound cardol isolated from the propolis of *T. incisa* also induces the death of SW620 cells via apoptosis through the activation of the mitochondrial pathway as evidenced by the increased expression of the active forms of caspases 9 and 3, poly (ADP-ribose) polymerase (PARP) cleavage, and depolarization of the mitochondrial membrane [71]. The authors also showed that cardol stimulated an increase in ROS levels, a common mechanism of the intrinsic apoptosis pathway, and cell death was partially reversed in the presence of the inhibitor N-acetyl-L-cysteine (NAC). The mechanisms underlying the cytotoxicity of the propolis extracts are shown in Figure 2.

## 7. Effects of Propolis on the Cell Cycle of Cancer Cell Lines

In addition to the loss of death mechanisms, cancer cells also undergo deregulation of their cell cycle, resulting in increased cell proliferation. In general, to proliferate, cells duplicate their contents and subsequently enter division in a process called the cell cycle [104].

Basically, the progression of this cycle is linked to specific phases that occur in the interphase and mitosis periods [105]. After leaving the G0 phase (quiescence), the cells enter interphase, which is sequentially divided into G1 (cell growth), S (DNA synthesis), and G2 (preparation for mitosis) phases, and then, the mitosis period or M phase, where the equal distribution of genetic material and cytokinesis, occurs [17, 106, 107].

The progression of the cell cycle is regulated by the joint action of cyclin-dependent kinases (CDKs) and proteins that control their activation and substrate specificity, which are called cyclins [108]. As members of a serine/threonine kinase family, CDKs are enzymatically active and participate in the following processes: (I) interaction with cyclins to form active heterodimeric complexes and (II) phosphorylation of threonine residues present in their activation segment [105, 109].

In mammals, the main CDKs involved in the cell cycle are CDK1, CDK2, CDK4, and CDK6, which form active enzyme complexes with specific cyclins: CDK1 with cyclins A/B, CDK2 with cyclins A/E, and CDK 4/6 with cyclin D [110]. CDKs 4/6 and cyclin D act in G1-S phases of the cell cycle, preparing cells for the S phase, where CDK 2 and cyclins A/E participate at its onset and progression, while CDK 1 and cyclins A/B mediate the S-G2 and G2-M transitions [109, 111].

The activity of CDKs that contribute to advancement of the cell cycle is induced by mitogenic signals and inhibited by activation of cell cycle checkpoints, which respond to genetic integrity failures [107, 112]. In addition, the progression of the cell cycle is blocked by the tumor-suppressive action of retinoblastoma (pRb) and p53 proteins [105, 113] and the action of endogenous inhibitors of cyclin-dependent kinases (CKIs), which control CDK activity [114].

Humans express two families of CKIs: the lnk family (p15, p16, p18, and p19), which binds to CDKs 4/6 and prevents their interaction and activation by cyclin D, and the Cip/kip family (p21, p27, and p57), which interrupts and inactivates CDK/cyclin complexes, such as CDK2/cyclin A or E and CDK1/cyclin A or B [114–116]. In general, CKIs promote cell cycle arrest [105] and play a critical role in cell cycle regulation [117]. Thus, before returning to the division process, possible damage to the genetic material can be repaired, or severely damaged cells are eliminated via apoptosis [115, 118].

The cell cycle is a highly regulated and ordered process due to rigorous cooperation between CDKs, cyclins, and CKIs [119]. However, in cancer cells, the levels of these proteins are often deregulated due to mutations and abnormal expression of cell cycle genes [105, 120]. These mutations inactivate CKIs and hyperactivate CDKs [118]. Upon the deactivation of its inhibitory mechanisms, the cell cycle and proliferation occur uncontrollably [105]. Thus, CDK inhibitors are an alternative treatment for cancer, and one of the focuses in related research is the development of drugs targeting the cell cycle and CDK transcription [121].

**Figure 3:**
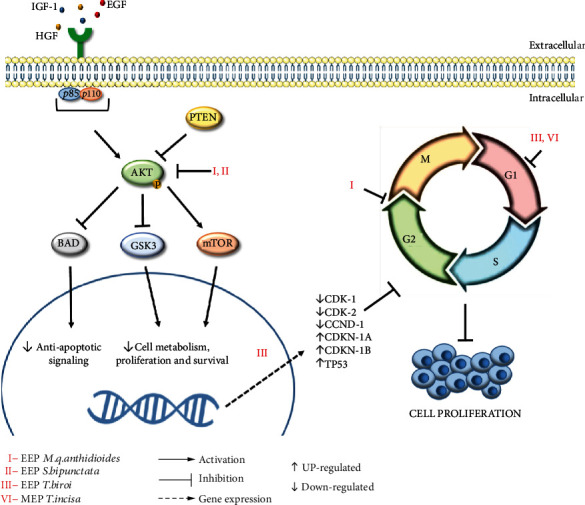
Modulation of the P13K/AKT signaling pathway and cell cycle arrest promoted by propolis extracts from stingless bees.

In recent decades, the action of various compounds derived from natural products in regulating the expression of proteins involved in cell cycle modulation has received considerable attention [122]. Recently, studies involving the propolis of some species of stingless bees have attracted attention given the promising effects of this natural product on cancer cells, as summarized in Table 3 and represented in Figure 3.

The propolis produced by *T. incisa* promotes cell cycle arrest in SW620 cells in the G1 phase [100], while the propolis produced by *M. q. anthidioides* induces cell cycle arrest in SK-MEL-28 cells in the G2/M phase [57]. Desamero *et al*. [16] highlighted that the propolis of the species *T. biroi* arrests the cell cycle of gastric cancer cell lines in the G0/G1 phase through the positive modulation of the expression levels of genes encoding cell cycle inhibitor proteins, such as CDKN1A (*Cyclin-Dependent Kinase Inhibitor 1A*) and CDKN1B (*Cyclin-Dependent Kinase Inhibitor 1B*) and a tumor suppression gene (TP53).

The TP53 gene encodes p53, a protein involved in DNA repair, cell cycle arrest, and apoptosis [123]. Compared to all other human genes, TP53 has the highest mutation rate [124]. Additionally, Desamero *et al*. [16] found that the ethanolic extract of *T. biroi* propolis downregulates the expression levels of the CDK1, CDK2, and CCND1 genes, which encode the CDK1, CDK2, and cyclin D1 proteins

In this context, as CDKs are usually hyperactivated in cancer, these molecules are one of the key targets for controlling the progression of this disease [125], highlighting the little-explored pharmacological potential of propolis from stingless bees.

## 8. Effects of Propolis on Intracellular Signaling Pathways

Intercellular communication is performed by small molecules (growth factors, hormones, cytokines, and neurotrophic factors) and other extracellular signaling molecules that interact with specialized “targets” (receptors) in the cell membrane, inducing a series of intracellular processes [126, 127]. Receptor tyrosine kinases (RTKs) function by activating and regulating signaling pathways related to various cellular processes, such as cell growth, proliferation, differentiation, survival, gene transcription, and metabolic regulation [128].

Several diseases result from genetic mutations or abnormalities that can alter the activity, quantity, cellular distribution, or regulation of RTKs; for example, mutations in these receptors and exacerbated activation of their signaling pathways have been associated with diabetes, inflammation, and cancer [129]. Signaling pathways such as RAS and PI3K can also be affected by mutations and contribute to the development of cancer in humans [130].

One of the most frequently altered pathways in human cancers is the PI3K signaling pathway [131]. Alterations include losses of lipid phosphatases (PTEN and INPP4B) and mutations in genes encoding (I) catalytic subunits (p110*α* and p110*β*), (II) regulatory subunits (p85*α*, p55*α*, and p50*α*), (III) PI3K activator K-RAS, and (IV) AKT (effector protein of the PI3K pathway) and its isoforms (AKT1, AKT2, and AKT3) [132, 133]. Total phosphorylation of AKT activates a multitude of downstream targets; through phosphorylation of its various substrates (mTOR, GSK3, NF-*κ*B, MDM2, BAD, and FKHR), AKT exerts signals that lead to cell growth and differentiation, angiogenesis, and apoptosis inhibition [130, 134] and are related to the onset of metastases [135].

Several efforts have been made to develop effective therapies targeting PI3K/AKT signaling; however, current PI3K and AKT inhibitors have shown limited efficacy and restricted doses due to toxicity [131, 132]. In this context, the search for new agents that can act on different signaling pathways, including PI3K/AKT, is key for the development of new therapies that can improve the quality of life of cancer patients.

Among the several studies on propolis from stingless bees with anticancer potential addressed in this review, only Cisilotto *et al.* [57] further investigated the effects of propolis from *S. bipunctata* and *M. q. anthidioides* on PI3K/AKT, a signaling pathway frequently altered in human cancers. They showed that the propolis of the two stingless bee species decreased the levels of protein expression, including one of the effector isoforms of the PI3K signaling pathway, as shown in Figure 3. The decreased signaling may be a mechanism related to other findings of the study, such as decreased levels of BCL-2 proteins, cell death by apoptosis, and decreased migration and invasion of human melanoma cells (SK-MEL-28).

## 9. Antioxidant and Anti-inflammatory Activities of Propolis: Chemopreventive Potential

Several pathophysiological conditions are directly related to the development of different types of cancer, including oxidative stress, chronic inflammation, and prolonged exposure to mutagens. Propolis from stingless bees has considerable potential to prevent deleterious changes in cellular metabolism since its antioxidant, anti-inflammatory, and immunomodulatory potential has already been described [42, 43, 60].

Antioxidant substances can inhibit and/or reduce the damage caused by reactive species by minimizing their reaction with biomolecules, such as proteins, lipids, and nucleic acids [136]. At normal physiological levels, reactive oxygen species (ROS) or reactive nitrogen species (RNS) play vital functions during cellular respiration, phagocytosis, inflammation, platelet aggregation, and angiogenesis [137]. However, excess radical species in the body, especially hydroxyl ^·^OH radicals, are related to the activation of different oncogenes or mutations of tumor suppression genes, which are important steps in the development of cancer [138].

The relationship between reactive species and chronic inflammatory processes is directly related to carcinogenesis: (I) high ROS concentrations alter membrane permeability, modify proteins, decrease the catalytic activity of enzymes, generate DNA damage, and consequently lead to genomic instability [139]; and (II) chronic inflammatory processes may play a critical role in cancer initiation, development, growth, and metastasis [140], since cytokines regulate cell proliferation, survival, differentiation, migration, and death [141].

Propolis from stingless bees has already been described as having antioxidant potential, acting in the direct scavenging of free radicals and the inhibition of lipid peroxidation [36, 58, 77, 142]. Lipid peroxidation results from reactive species attacking lipids present in the cell plasma membrane, promoting the production of highly carcinogenic molecules in human cells, such as malondialdehyde, which leads to insertions, deletions, and substitutions of base pairs in DNA [143].

Lipid peroxidation in nontumor cells may also be due to inflammatory responses associated with the development and invasion of cancer [138]. The relationship between reactive species and chronic inflammatory processes is directly related to carcinogenesis; therefore, compounds that neutralize both deleterious effects of the tumor microenvironment are of great importance [144, 145]. Campos *et al.* [55] noted that in addition to acting as an antitumor agent in a leukemic cell line, the ethanolic extract of propolis of *T. fiebrigi* has antioxidant activity by inhibiting lipid peroxidation in human erythrocytes and anti-inflammatory activity by inhibiting hyaluronidase. Similarly, Massaro *et al*. [54] demonstrated that propolis of the Australian species *Tetragonula carbonaria* inhibits the activity of the enzyme 5-lipoxygenase (5-LOX), which is known to catalyze the production of proinflammatory mediators. In addition, polar extracts of *T. carbonaria* propolis have been described to have antioxidant properties and to suppress production of the proinflammatory eicosanoid leukotriene B4 in human neutrophils stimulated with ionomycin [146].

Substances found in propolis of stingless bees, such as quercetin and apigenin, promote downregulation of toll-like receptor 4 (TLR4) and a decline in protein levels of AP-1 (key transcription factor which regulates several cytological processes, including proliferation, apoptosis, and cell migration) and together with the increase in the sirtuin 1(Sirt1)/Nrf2 pathway induce antioxidant defenses and reduce inflammation and oxidative stress [147].

## 10. Selectivity and Safety of Propolis Use

Despite the benefits promoted by drugs in controlling cancer cell proliferation, chemotherapy drugs have important side effects, such as fatigue, diarrhea, nausea, hair loss, cardiovascular and renal complications, and chronic effects such as infertility [148]. Thus, the search for compounds or molecules that have antitumor selectivity and minimal side effects is encouraged. Studies show that propolis from different species of stingless bees has selectivity against tumor cell lines, resulting in higher cytotoxicity than that observed in nontumor lines [68, 100].

Bonamigo *et al.* [77] found that the ethanolic extract of propolis of *Plebeia droryana* promotes lower cytotoxicity against human peripheral blood mononuclear cells than K562 cells. The erythroleukemic cell line K562 is described for its resistance to apoptotic death [149]; therefore, other types of death, such as necrosis or necroptosis, are necessary to inhibit the proliferation of these tumor cells [83]. Propolis of the species found in the Central-West region of Brazil, including *M. orbignyi* [58], *T. fiebrigi* [55], *S. depilis*, and *M. q. anthidioides* [36], also promotes the death of this leukemic cell line that is resistant to conventional chemotherapeutic agents.

In a study by Cisilotto *et al*. [57], when evaluating the cytotoxicity of *S. bipunctata* and *M. q. anthidioides* in a human melanoma cell line (SK-MEL-28) and a human melanocyte nontumor cell line (NGM), the authors found high selectivity for tumor cells. Additionally, these propolis extracts potentiated the action of the chemotherapeutic agent vemurafenib, which is used in cases of metastatic melanoma with mutations in the BRAF protooncogene. Mohamed *et al*. [76] investigated the selectivity of the cytotoxic action of the propolis extract of *Tetrigona apicalis* in a breast cancer cell line (MCF-7) and an epithelial breast nontumor cell line (MCF 10A) and found that after 72 h of incubation, the tumor cells were more sensitive to treatment.

In addition to evaluating selectivity in *in vitro* cell models, selectivity must also be assessed in multicellular *in vivo* models [42], which is a key step in the development of new drugs. From this perspective, 21 samples of propolis from Brazilian stingless bees were analyzed for toxicity in the microcrustacean *Artemia salina*, which demonstrated lethal doses in 50% of the individuals (LD50) ranging from 0.3 ± 0.2 to >1000 *μ*g/mL [150].

Bonamigo *et al.* [36] investigated the activity of the ethanolic extract of propolis from *S. depilis* and *M. q. anthidioides* (250-1000 *μ*g/mL) in the *Caenorhabditis elegans* animal model and observed no toxic or lethal effects after 24 h of incubation despite this extract showing effective antitumor activity. The nematode *C. elegans* has been widely used in toxicity studies of pharmacological compounds, including natural products [151, 152]. *C. elegans* is an excellent *in vivo* model to complement cell culture assays, has a good correlation with the LD50 observed in rodents, and contains many genes and signaling pathways similar to those in humans [151].

However, studies on the toxicity or pharmacological potential of propolis from stingless bees in mammals are still scarce. Recently, Desamero *et al*. [16] investigated the antitumor potential of *T. biroi* propolis (100 mg/kg) in A4gnt-knockout (KO) mice (gastric adenocarcinoma model) and its effects on a C57BL/6 J model (wild-type). The authors demonstrated the efficacy of the extract on the regression of histological and macroscopic lesions of pyloric gastric tumors; in the control animal model, no differences were observed in the morphology or thickness of the gastric mucosa or T-lymphocyte infiltration between animals treated with distilled water or the ethanolic extract of *T. biroi*.

As a means to complement *in vitro* and *in vivo* studies on the toxicity of propolis extracts and their compounds, Lavinas *et al*. [42] analyzed the potential toxicity of these products in silico using ADMET Predictor^™^ software and found low toxicity, indicating that these compounds are potentially safe to use. Recent technological and scientific advances have contributed to the natural product-based drug discovery, such as tools that enable the analysis of genetic information, the prediction of chemical structures and pharmacological activities, and the integration of data sets with diverse information [153]

However, Lavinas *et al*. [42] emphasized the need for preclinical studies to demonstrate the efficacy of propolis in the development of future drugs. Recently, there are no clinical studies conducted with propolis of stingless bees; in addition, Chiu et al. [29] highlight that few clinical trials are conducted with propolis of the species *A. mellifera* and its active components, which present unsatisfactory results. The authors attribute the reduced efficacy of propolis in clinical trials to its low solubility in water and suggest the use of formulations in nanoparticles. Natural product-based nanoformulations have been proposed to solve problems such as the low solubility of these compounds in water, in addition to minimizing side effects of therapy against different types of cancers [154].

Khongkaew and Chaemsawang [155] evaluated the propolis of stingless bees in the formulation of nanoparticles and reported that it presents high physicochemical stability, besides not presenting toxicity in human fibroblast cells, and suggests future pharmaceutical applications.

## 11. Conclusions and Perspectives

Cancer is a highly complex disease in which alterations can occur in different genes, consequently altering multiple cells signaling pathways in different types of tumors. Most chemical treatments targeting cancer are not very selective and cause complications for patients and/or are very expensive. Therefore, research on natural products such as propolis is extremely relevant to identify new therapies that minimize side effects, increase the effectiveness of current treatments, and enable the development of new drugs with more effective mechanisms of action.

The scenario presented regarding the antitumor activity of propolis from stingless bees, as well as its mechanisms of action and induced cell death profile, demonstrates the substantial bioprospecting potential of this natural product. Given the diversity of stingless bee species distributed around the world, much remains to be investigated in the search for new prototypes for the development of anticancer drugs. However, with the accelerated loss of biodiversity due to anthropogenic activity, promoting the preservation of these species and their environments, as well as research on the antitumor activity of their bee products, is an urgent endeavor.

Newman and Cragg [2] emphasize that natural products are still the best sources for the discovery of novel agents/active templates and offer the potential to discover novel structures that can lead to effective agents in a variety of human diseases, including cancer. In summary, the chemical diversity and different mechanisms of cell death induced by propolis from stingless bees reflect the promising potential of this product for the development of new antitumor drugs, which should be expanded through further preclinical and clinical studies.

## Figures and Tables

**Table 1:** Chemical compounds and anticancer potential of propolis from species of stingless bees in different cancer lineages.

Stingless bee species	Geographic origin	Type of extract	Compounds identified	Anticancer potential	Cell lineage	References
*Trigona laeviceps*	Samut Songkhram Province, Thailand	Ethanolic (fractioned) (methanol and dichloromethane)	NS	Antiproliferative	Ductal carcinoma (BT474) Lung cancer (ChaGo-I) Colon adenocarcinoma (SW620) Liver cancer (HepG2) Gastric cancer (KATO-III)	[68]
*Trigona sp.*	Maharashtra, India	Hydroethanolic Ethanol/water 70/30 %	NS	Cytotoxic	Human breast adenocarcinoma (MCF-7) Human colon adenocarcinomas (HT-29 and Caco-2) Murine melanoma (B16F1)	[56]
*Trigona incisa* *Timia apicalis* *Trigona fusco-balteata* *Trigona fuscibasis*	Kalimantan, Indonesia	Methanolic Fractioned (ethyl acetate and hexane)	NS	Cytotoxic	Ductal carcinoma (BT474) Lung cancer (ChaGo-I) Colon adenocarcinoma (SW620) Liver cancer (HepG2) Gastric cancer (KATO-III)	[69]
Melipona orbignyi	Mato Grosso do Sul, Brazil	Hydroethanolic Ethanol/water 80/20 %	Aromatic acids, alcohols, terpenes, phenolic acids, flavonoids, and sugars	Cytotoxic	Human leukemia (K562)	[58]
*Tetragonisca fiebrigi*	Mato Grosso do Sul, Brazil	Hydroethanolic Ethanol/water 80/20 %	Benzoic and kaurenoic acids, sugars, retinol, tocopherols, cinnamic acids	Cytotoxic	Human leukemia (K562)	[55]
Tetragonula iridipennis	Pudukkottai District, India	Hydroethanolic Ethanol/water 70/30 %	NS	Cytotoxic	Human lung cancer (A549)	[70]
*Scaptotrigona depilis*	Mato Grosso do Sul, Brazil	Hydroethanolic Ethanol/water 80/20 %	*β*-Sitosterol, *β*-amyrin, *α*-amyrin, *β*-amyrin acetate, tocopherol, vanillin acid	Cytotoxic	Human leukemia (K562)	[36]
*Melipona quadrifasciata anthidioides*	Mato Grosso do Sul, Brazil	Hydroethanolic Ethanol/water 80/20 %	Stigmasterol, taraxasterol, *β*-sitosterol, *β*-amyrin, *α*-amyrin, *β*-amyrin acetate, tocopherol, vanillic acid, caffeic acid, quercetin, luteolin, apigenin	Cytotoxic	Human leukemia (K562)	[36]
*Plebeia droryana*	Mato Grosso do Sul, Brazil	Hydroethanolic Ethanol/water 80/20 %	*β*-Sitosterol, *β*-amyrin, *α*-amyrin, *β*-amyrin acetate, tocopherol	Cytotoxic	Human leukemia (K562)	[65]
*Trigona incisa*	Kalimantan, Indonesia	Methanolic Fractioned (ethyl acetate and hexane)	Isolated compound Cardol (5-pentadecyl resorcinol)	Cytotoxic, antiproliferative	Colon adenocarcinoma (SW620)	[71]
*Trigona minor*	Ben Tre Province, Vietnam	Ethanolic (fractioned) (chloroform, ethyl acetate, and hexane)	Lanostane-type triterpenoid, cycloartane-type triterpenoids, 23-hydroxyisomangiferolic acid B, and 7-hydroxyisomangiferolic acid	Cytotoxic	Human pancreatic cancer (PANC-1)	[72]
*Trigona sirindhornae*	Chanthaburi Province, Thailand	Ethanolic (fractioned) (methanol and dichloromethane)	NS	Cytotoxic	Human head and neck cancer (HN30)	[37]
*Tetragonula pagdeni*	Chanthaburi Province, Thailand	Methanolic	Xanthones (*α*-mangostin and *γ*-mangostin)	Cytotoxic	Colon adenocarcinoma (Caco-2) Human melanoma (SK-MEL-28) Liver cancer (Hep-G2) Papilloma carcinoma (KB)	[37]
*Lepidotrigona ventralis* *Lepidotrigona terminata*	Chanthaburi Province, Thailand	Methanolic	NS	Cytotoxic	Colon adenocarcinoma (Caco-2) Human melanoma (SK-MEL-28) Liver cancer (Hep-G2) Papilloma carcinoma (KB)	[37]
Scaptotrigona bipunctata	Santa Catarina, Brazil	Hydroethanolic Ethanol/water 70/30 %	Piperidinic alkaloids, flavones, triterpenes	Cytotoxic, antimigratory, anti-invasion	Human melanoma (SK-MEL-28)	[57]
Melipona quadrifasciata anthidioides	Santa Catarina, Brazil	Hydroethanolic Ethanol/water 70/30 %	Polyphenols, flavonoids (7-O-aromadendrin, naringenin), p-coumaric acid, pinusenocarp, mepuberin	Cytotoxic, Antimigratory, Anti-invasion	Human melanoma (SK-MEL-28)	[57]
*Heterotrigona itama*	Terengganu and Kelantan, Malaysia	Ethanolic	NS	Cytotoxic	Human cervical carcinoma (HeLa)	[73]
*Tetragonula biroi*	Philippines	Ethanolic	Carbohydrates, steroids, alkaloids, anthraquinones, phenols, terpenoids	Cytotoxic, antiproliferative	Gastric cancer (AGS, MKN-45, NUGC-4, MKN-74)	[16]
*Trigona sp*.	South Sulawesi, Indonesia	Ethanolic	Diterpenes, sesquiterpenes, actinopyrones (not confirmed)	Cytotoxic, antiangiogenic	Human breast cancer (MDA-MB-231)	[74]
*Trigona* spp.	South Sulawesi, Indonesia	Hydroethanolic Ethanol/water 70/30 %	NS	Cytotoxic, antiproliferative	Breast cancer cell line (MCF-7)	[75]
*Tetrigona apicalis*	Malaysia	Hydroethanolic Ethanol/water 80/20 %	Sesquiterpenes (*β*-caryophyllene, copaene), triterpenoids (*β*-amyrin, *α*-amyrin)	Cytotoxic	Breast cancer cell line (MCF-7)	[76]

NS: not studied.

**Table 2:** Cell death profile and mechanisms of action induced by propolis produced by species of stingless bee on different cancer lineages.

Stingless bee species	Type of extract or isolated compound	Cell lineage	Assays	Types of cell death	Mechanisms of action	References
*Trigona sp.*	Hydroethanolic Ethanol/water 70/30 %	Human colon adenocarcinomas (HT-29 and Caco-2) Murine melanoma (B16F1)	Flow cytometer	Apoptosis	NS	[56]
Melipona orbignyi	Hydroethanolic Ethanol/water 80/20 %	Human leukemia (K562)	Flow cytometer	Necrosis	NS	[58]
*Tetragonisca fiebrigi*	Hydroethanolic Ethanol/water 80/20 %	Human leukemia (K562)	Flow cytometer	Necrosis^∗^ and apoptosis^∗∗^	NS	[55]
*Trigona incisa*	Methanolic Fractioned (ethyl acetate and hexane)	Colon adenocarcinoma (SW620)	Flow cytometer	Apoptosis	NS	[100]
*Scaptotrigona depilis* *Melipona quadrifasciata anthidioides*	Hydroethanolic Ethanol/water 80/20 %	Human leukemia (K562)	Flow cytometer	Necrosis^∗^ and apoptosis^∗∗^	NS	[36]
*Plebeia droryana*	Hydroethanolic Ethanol/water 80/20 %	Human leukemia (K562)	Flow cytometer	Necroptosis^∗^ and apoptosis^∗∗^	Activation of RIPK1 and increased caspase 3	[77]
*Trigona incisa*	Cardol (5-pentadecyl resorcinol)	Colon adenocarcinoma (SW620)	Caspase activity, mitochondrial membrane potential, Western blotting	Apoptosis	Increased activity of caspases 3 and 9, PARP cleavage Mitochondrial depolarization ROS increase Partial reversal of apoptosis with NAC	[71]
Scaptotrigona bipunctata Melipona quadrifasciata anthidioides	Hydroethanolic Ethanol/water 70/30 %	Human melanoma (SK-MEL-28)	Flow cytometer Western blotting	Apoptosis	ROS increase Reduction of mitochondrial membrane potential Decrease in BLC-2	[57]
*Heterotrigona itama*	Ethanolic	Human cervical carcinoma (HeLa)	Flow cytometer	Apoptosis	NS	[73]
*Tetragonula biroi*	Ethanolic	Gastric cancer (AGS, MKN-45, NUGC-4)	Flow cytometer qRT-PCR TUNEL assay	Apoptosis	Positive modulation of BAX and BAD transcription and negative modulation of BCL2L1 and BCL-2	[16]
*Trigona* spp.	Hydroethanolic Ethanol/water 70/30 %	Breast cancer cell line (MCF7)	Flow cytometer	Apoptosis	NS	[75]

NS: not studied. ^∗^Mainly. ^∗∗^Partially.

**Table 3:** *In vitro* effects of propolis from different stingless bee species on the cell cycle of cancer lineages.

Stingless bee species	Region/country	Extract type or isolated compound	Cell lineage	Assays	Effects on cell cycle	Involved mechanisms	References
*Trigona incisa*	Kalimantan, Indonesia	Methanolic Fractioned (ethyl acetate and hexane)	Colon adenocarcinoma (SW620)	Flow cytometer	Cell cycle arrest (G1 phase)	NS	[100]
Melipona quadrifasciata anthidioides	Santa Catarina, Brazil	Hydroethanolic Ethanol/water 70/30 %	Human melanoma (SK-MEL-28)	Flow cytometer	Cell cycle arrest (G2/M phases)	NS	[57]
*Tetragonula biroi*	Philippines	Ethanolic	Gastric cancer (AGS, MKN-45, NUGC-4)	Flow cytometer qRT-PCR	Cell cycle arrest (G0/G1 phases)	Positive modulation of the transcription of inhibitory genes of the cell cycle (CDKN1A, CDKN1B, tp53) and negative modulation for transcription of (CDK1, CDK2, and CCND1) related to kinases and cyclins	[16]

NS: not studied.
